# Identification of Key Influencers for Secondary Distribution of HIV Self-Testing Kits Among Chinese Men Who Have Sex With Men: Development of an Ensemble Machine Learning Approach

**DOI:** 10.2196/37719

**Published:** 2023-11-23

**Authors:** Fengshi Jing, Yang Ye, Yi Zhou, Yuxin Ni, Xumeng Yan, Ying Lu, Jason Ong, Joseph D Tucker, Dan Wu, Yuan Xiong, Chen Xu, Xi He, Shanzi Huang, Xiaofeng Li, Hongbo Jiang, Cheng Wang, Wencan Dai, Liqun Huang, Wenhua Mei, Weibin Cheng, Qingpeng Zhang, Weiming Tang

**Affiliations:** 1 Institute for Healthcare Artificial Intelligence Application Guangdong Second Provincial General Hospital Guangzhou China; 2 Faculty of Data Science City University of Macau Macao Special Administrative Region China; 3 University of North Carolina at Chapel Hill Project-China Guangzhou China; 4 School of Data Science City University of Hong Kong Hong Kong Special Administrative Region China; 5 Center for Infectious Disease Modeling and Analysis Yale School of Public Health Yale University New Haven, CT United States; 6 Department of HIV Prevention Zhuhai Center for Diseases Control and Prevention Zhuhai China; 7 School of Public Health Boston University Boston, MA United States; 8 Fielding School of Public Health University of California Los Angeles Los Angeles, CA United States; 9 London School of Hygiene and Tropical Medicine London United Kingdom; 10 Melbourne Sexual Health Centre Melbourne Australia; 11 Division of Infectious Diseases School of Medicine University of North Carolina at Chapel Hill Chapel Hill, NC United States; 12 School of Public Health Nanjing Medical University Nanjing China; 13 School of Social Work Michigan State University East Lansing, MI United States; 14 Zhuhai Xutong Voluntary Services Center Zhuhai China; 15 Department of Epidemiology and Biostatistics School of Public Health Guangdong Pharmaceutical University Guangzhou China; 16 Dermatology Hospital of Southern Medical University Guangzhou China; 17 Institute of Data Science and Department of Pharmacology and Pharmacy The University of Hong Kong Hong Kong Special Administrative Region China

**Keywords:** HIV self-testing, machine learning, MSM, men who have sex with men, secondary distribution, key influencers identification

## Abstract

**Background:**

HIV self-testing (HIVST) has been rapidly scaled up and additional strategies further expand testing uptake. Secondary distribution involves people (defined as “indexes”) applying for multiple kits and subsequently sharing them with people (defined as “alters”) in their social networks. However, identifying key influencers is difficult.

**Objective:**

This study aimed to develop an innovative ensemble machine learning approach to identify key influencers among Chinese men who have sex with men (MSM) for secondary distribution of HIVST kits.

**Methods:**

We defined three types of key influencers: (1) key distributors who can distribute more kits, (2) key promoters who can contribute to finding first-time testing alters, and (3) key detectors who can help to find positive alters. Four machine learning models (logistic regression, support vector machine, decision tree, and random forest) were trained to identify key influencers. An ensemble learning algorithm was adopted to combine these 4 models. For comparison with our machine learning models, self-evaluated leadership scales were used as the human identification approach. Four metrics for performance evaluation, including accuracy, precision, recall, and *F*_1_-score, were used to evaluate the machine learning models and the human identification approach. Simulation experiments were carried out to validate our approach.

**Results:**

We included 309 indexes (our sample size) who were eligible and applied for multiple test kits; they distributed these kits to 269 alters. We compared the performance of the machine learning classification and ensemble learning models with that of the human identification approach based on leadership self-evaluated scales in terms of the 2 nearest cutoffs. Our approach outperformed human identification (based on the cutoff of the self-reported scales), exceeding by an average accuracy of 11.0%, could distribute 18.2% (95% CI 9.9%-26.5%) more kits, and find 13.6% (95% CI 1.9%-25.3%) more first-time testing alters and 12.0% (95% CI –14.7% to 38.7%) more positive-testing alters. Our approach could also increase the simulated intervention’s efficiency by 17.7% (95% CI –3.5% to 38.8%) compared to that of human identification.

**Conclusions:**

We built machine learning models to identify key influencers among Chinese MSM who were more likely to engage in secondary distribution of HIVST kits.

**Trial Registration:**

Chinese Clinical Trial Registry (ChiCTR) ChiCTR1900025433; https://www.chictr.org.cn/showproj.html?proj=42001

## Introduction

Men who have sex with men (MSM) have a higher burden of HIV [1]. In China, HIV prevalence among MSM is 6.3% in 2019 [2]. However, over 40% of Chinese MSM have never been tested [3], and over 30% of MSM living with HIV do not know their serostatus [4]. More efficient case-finding for undiagnosed people living with HIV and starting treatment is essential for HIV control [5]. To increase the coverage of HIV testing, HIV self-testing (HIVST) has been recommended by the World Health Organization (WHO) [6], which has high acceptability among MSM [7].

Secondary distribution is one of the novel ways to increase the use of HIVST [8]. Within this service delivery model, individuals, commonly referred to as “indexes,” take the initiative to request and receive multiple HIVST kits. Subsequently, they play a crucial role in distributing these HIVST kits to individuals within their social network. These network members, specifically sexual partners and close associates within the MSM community, are designated as “alters” [9,10]. In essence, indexes serve as the primary recipients and distributors of the HIVST kits, while alters represent the recipients of these kits within the social circle. Such a strategy could significantly improve HIV testing coverage by reaching people who have limited access to HIV testing and potentially detect more undiagnosed people with HIV [10]. To further expand the use of this strategy and enhance the efficiency of distribution, it could be useful to identify influential indexes who are more likely to distribute kits to more alters (eg, ≥2 alters), people living with HIV who are undiagnosed, or first-time testers.

However, existing methods for identifying MSM key influencers are limited in the following 2 respects. First, some studies selected key influential people based on human intuition and then trained them as opinion leaders [11,12]. This selection process of key influencers lacks a scientific basis and is not reliable or generalizable [13]. Second, other studies used self-reported leadership scales, such as those among drug users [14]. This method is more scientific because of self-reported leadership scales, but it is still relatively subjective. Even if all self-reported leadership items were reliable and valid, these identified leaders in the community might not be key influencers for secondary distribution of HIVST kits.

Artificial intelligence (AI), including machine learning (ML) approaches, is a promising method to identify key influencers [15,16]. In the area of HIV intervention, ML models also performed well in different kinds of key population classification tasks, such as identifying people at a relatively higher risk of HIV [17] and identifying suitable candidates for pre-exposure prophylaxis (PrEP) [18]. Thus, ML approaches have potential to be used for identifying key influencers for secondary distribution of HIVST kits.

**Figure 1:**
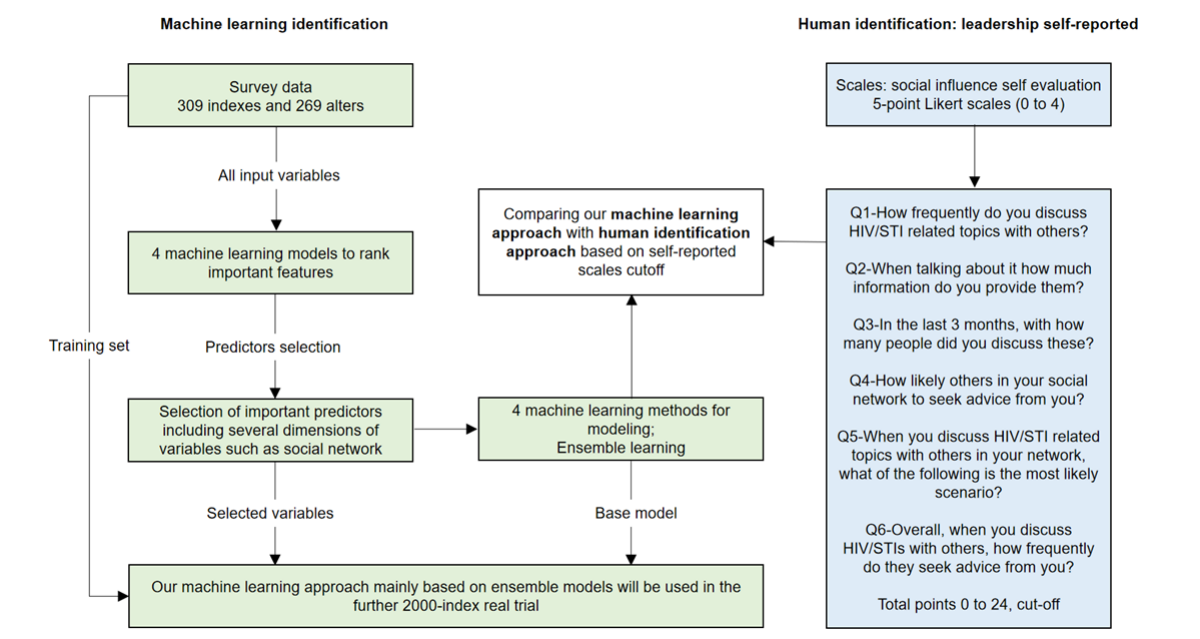
Framework of our study.

Using data collected from previous studies [19], we propose a novel ensemble ML approach (Figure 1) to identify key influencers for secondary distribution of HIVST kits where indexes applied for testing kits for distribution, while alters were those individuals who received these kits. Specifically, our ML models were trained to obey 3 rules to identify key influencers: key-distribution influencers (ie, key distributors) who are more likely to distribute kits to as many alters as possible (eg, no fewer than 2 kits in 10 months), key-promotion influencers (ie, key promoters) who contribute to promoting first-time testing among alters, and key-detection influencers (ie, key detectors) who distribute kits to alters who are undiagnosed people living with HIV.

## Methods

### Data Processing

The data set was derived from a 3-arm randomized controlled trial of secondary distribution of HIVST kits in Zhuhai, China [19]. This trial was registered with the Chinese Clinical Trial Registry (ChiCTR1900025433). All participants gave digital written informed consent by providing electronic signatures before the taking the web-based baseline survey. Between October 21, 2019, and September 14, 2020, a 3-arm randomized controlled, single-blinded trial was conducted on the web among 309 individuals (defined as “index participants”) who were assigned male at birth, aged 18 years or older, ever had male-to-male sex, willing to order HIVST kits on the internet, and consented to take surveys on the web. In this trial, 309 MSM were randomly assigned to the control group (standard secondary distribution [SD] arm; SD group), the intervention I group (SD with monetary incentives [SD-M] arm; SD-M group), or the intervention II group (SD-M and peer referral [SD-M-PR] arm; SD-M-PR group). Monetary incentives implies that the index participants in the SD-M and SD-M-PR groups could receive a fixed incentive of US $3 on the web for a verified test result uploaded to the digital platform by each unique alter. Monetary incentives and peer referral implies that the index participants in the SD-M-PR group could additionally have a personalized peer referral link for alters to order kits on the web as an intervention strategy. Of 309 indexes, 60 were key distributors who passed the kits to at least 2 alters. Additionally, there were 73 key promoters, leading to 103 alters who were first-time testers; and 23 key detectors, leading to 25 alters who were undiagnosed people living with HIV, as defined above. The trial profile infographic with more details can be found in Zhou et al [20].

### ML Modeling

We formulated a strategy to identify key influencers as a binary classification problem, and 4 ML models were constructed, including logistic regression, support vector machine, decision tree, and random forest [21,22]. Each model has its pros and cons (Multimedia Appendix 1) in performing the classification task; hence, we comprehensively combined the predictions of all 4 models to mitigate overfitting and model biases by adopting an ensemble learning approach [23], which could synthesize the strengths from each ML model. Specifically, we used the voting classifier in soft mode as the ensemble method, considering the probabilities yielded by each ML model, and these probabilities would be weighted and averaged; consequently, the winning class would be the one with the highest weighted and averaged probability.

To evaluate ML models for such classification tasks, we used 4 metrics for performance evaluation: accuracy, precision, recall, and *F*_1_-score (Multimedia Appendix 2). Accuracy is defined as a ratio of correctly predicted observations to the total number of observations. The *F*_1_-score takes both the precision and the recall into consideration and is defined as the harmonic mean of precision and recall:



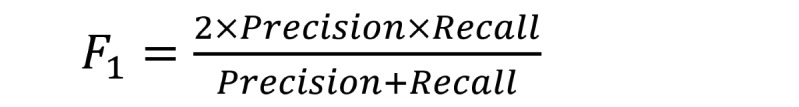



We used 5-fold cross-validation [24] to ensure the robustness of the models and compared the average values of each metric. Specifically, we randomly sampled 80% of the data for training and 20% for testing. Experiments for each metric were repeated 5 times as every time 1 fold (ie, 20% of the data) would constitute the testing set and the remaining 4 folds (ie, 80% of the data) would be trained for model construction and parameter learning. The final average values of each metric are the average performance of the 5 folds’ testing set.

### Selection of Predictors

First, we incorporated original predictors (ie, input variables from the survey) into our classification models using the aforementioned 4 ML models. Then, we obtained a predictor ranking list of each ML model ordered by the importance of every variable (Multimedia Appendix 3). The 4 ML models voted for the final selected predictors, which ranked at the top in all 4 importance ranking lists.

Specifically, the logistic regression model provided us with the coefficient of each predictor, while the other 3 models (support vector machine, decision tree, and random forest) provided the importance level. Specifically, in logistic regression, we ranked the importance of each characteristic in accordance with the absolute value of the standardized coefficient. For the support vector machine model, we adopted the Recursive Feature Elimination algorithm to generate a weighted vector when training, and then in each iteration, we eliminated a least important feature through the above-weighted vector. For decision tree and random forest models, the Gini index in the Classification and Regression Tree algorithm determined the importance of every variable.

### Identification System

After determining the top predictors by importance ranking, we ran the same 4 ML models based on these selected variables to check and obtain the classification performance. Then, we used ensemble learning to combine the findings from all ML models. The whole process, including ML modeling, predictor selection, and modeling based on selected important variables (ensemble learning), represents a novel intelligent identification system (illustrated in Figure 1), which can be adopted in the implementation program in the secondary distribution of HIVST kits in the future to identify key influencers among MSM.

We ran all ML experiments in Python (version 3.7; Python Software Foundation), and the code is available upon request from the corresponding author.

### Human Identification Approach

For comparison with our findings using ML models, we used 2 self-reported scales as the human identification approach. According to existing literature, self-evaluated leadership scales are commonly used to identify key influencers [11,12,14]. These self-reported leadership scales asked indexes to evaluate the likelihood of 6 social influence–related scenarios on a scale of 0 to 4, and the total points ranged from 0 to 24. Based on the previous literature, indexes (around 20% out of a total of 309 indexes) who distributed at least 2 kits were defined as key distributors on the ground truth. Hence, we set a cutoff of the top 60 indexes (which was also around 20%) by rank order in our 6-question self-reported scales (Figure 1) as the human-identified key influencers. However, 49 indexes received at least 11 points in the self-reported scales, while 81 indexes received at least 10 points. In other words, since the 49th to the 80th indexes received the same points in these self-reported scales, we were unable to determine exactly which of these indexes ranked in the top 60. As a result, we regarded these 2 scale cutoffs as human identification baselines together, recorded as cutoffs A and B, respectively.

### Simulation

Finally, we further conducted a simulation model to mimic the secondary distribution process on the MSM’s social network and to compare the intervention efficiency of identification of the key influencers by the ML models and by conventional human identification approaches. Here, intervention efficiency is defined as the number of individuals who have self-tested at the end of the simulation. Specifically, simulation technologies [25,26] on HIV-related networks [27,28] can also model distribution network characteristics.

We simulated the secondary distribution process on each test set of the 5-fold cross-validation. Given indexes in each test set, we constructed a network containing both indexes and alters who received self-testing kits from these indexes. There would be an edge between an index and an alter if the alter received a kit with the corresponding confirmation code of the index. Self-testing kits would be distributed through edges on the network. Specifically, we summarized and interpreted the secondary distribution process observed from our empirical studies [20,29] into a simplified diffusion model. We used a Poisson distribution to mimic the distribution behavior. Only 1 parameter is needed in the Poisson distribution: the mean number of HIVST kits an individual wants to distribute at each time step. We set it as the number of received HIVST kits. The number of HIVST kits allocated to indexes is set at 4, as the number of HIVST kits an index can order in our empirical experiments is generally no more than 5 [20]. The code for simulations is available upon request from the corresponding author.

### Ethical Considerations

Ethics approval of this trial was obtained through the Zhuhai Center for Disease Control and Prevention (ZhuhaiCDC-201901) [19]. All participants provided written informed consent.

## Results

### Modeling Results

We compared the performance of ML classification and ensemble learning with that of the human identification approach based on leadership self-evaluated scales in terms of the 2 nearest cutoffs. In our survey data, 60 (19.4%; ie, around 20%) indexes who distributed at least 2 kits were key distributors on the ground truth. In addition, these key distributors reached more than 70% of alters in total. Additionally, there were 73 key promoters who helped us promote first-time testing to 103 alters and 23 key detectors who helped us detect 25 positive alters.

Table 1 shows that ML classification significantly outperformed human identification cutoffs irrespective of the type (ie, key distributors, key promoters, and key detectors) adopted to define the key influencers (technical details provided in Multimedia Appendix 1). The model using ensemble learning also outperformed the human identification approach and nearly achieved the highest value among all models in terms of the performance metrics. Specifically, for 3 classification training rules (ie, 3 types of key influencers), the classification performance of ensemble learning obtained an accuracy of 90%, 93%, and 82% for key distributors, key promoters, and key detectors, respectively, all exceeding human identification (the cutoffs of the self-reported scales). Therefore, the ensemble learning approach combining 4 ML models could better capture key influencers, compared with the other approaches studied, with an 11.0% higher accuracy on average than human identification approaches.

**Table 1:** Machine learning classification results using 5-fold cross-validation.

Metrics	Key distributors; number of alters≥2			Key detectors; positive alters≥1			Key promoters; new-tester alters≥1	
	Accuracy	*F*_1_-score	Accuracy		*F*_1_-score	Accuracy		*F*_1_-score
LR^a^	0.89	0.93	0.92		0.95	0.83		0.89
SVM^b^	0.90	0.94	0.93		0.96	0.82		0.89
DT^c^	0.91	0.94	0.91		0.95	0.80		0.88
RF^d^	0.88	0.93	0.93		0.96	0.78		0.87
Ensemble^e^	0.90	0.94	0.93		0.96	0.82		0.89
Cutoff A^f^	0.72	0.82	0.85		0.88	0.68		0.78
Cutoff B^g^	0.79	0.87	0.88		0.91	0.73		0.83

^a^LR: logistic regression.

^b^SVM: support vector machine.

^c^DT: decision tree.

^d^RF: random forest.

^e^Ensemble: ensemble learning.

^f^Cutoff A: lower cutoff of the self-reported scales.

^g^Cutoff B: higher cutoff of the self-reported scales.

Table 2 compares the number of distributed kits from key influencers identified using the ensemble learning model and the human identification approach. Both the ensemble learning model and the human identification approach classified 49 key influencers each, but those identified using the ensemble learning approach distributed 146 kits, equating to 54% (146/269) of alters. In contrast, the 49 key influencers identified using the human identification approach only distributed 97 kits. In addition, the same 49 key influencers identified by the ensemble learning model identified 3 more people living with HIV and 14 more first-time testers than those identified using the human identification approach. In summary, our new approach could identify the distribution of 18.2% (95% CI 9.9%-26.5%) more kits, 13.6% (95% CI 1.9%-25.3%) more first-time testing alters, and 12.0% (95% CI –14.7% to 38.7%) more undiagnosed people living with HIV.

**Table 2:** Comparison among key influencers identified through ensemble learning^a^.

	Successfully distributed kits	Positive alters (ie, people living with HIV)	First-time testing alters
Machine learning identification, n	146	11	33
Self-reported scales, n	97	8	19
Total (original), n	269	25	103
Increased percentage, %	18.2	12.0	13.6

^a^The table shows the results obtained using the ensemble learning model for identifying key distributors, and this model happened to classify 49 key influencers (in 5-fold testing sets), sharing the same number with a certain scale’s cutoff. Therefore, our comparisons are rational. Such percentages and CIs are calculated on the basis of the total number (eg, if the total number of distributed kits is 269, the increased percentage of successfully distributed kits is calculated as [146–97]/269 rather than [146–97]/146).

### Simulation Results

We simulated the secondary distribution process on each test set of 5-fold cross-validation. The simulation results (Table 3) show that our ensemble ML approach could always obtain a higher intervention efficiency in each fold than the conventional human identification approach. Specifically, the average intervention efficiency of the ensemble ML model increased by 17.7% (95% CI –3.5% to 38.8%) compared to that of the self-reported scales cutoff method, which indicates a higher intervention efficiency of our novel method to identify key influencers.

**Table 3:** Simulation results of intervention efficiency after identification of key influencers.

Efficiency	Fold 1	Fold 2	Fold 3	Fold 4	Fold 5	Average
Ensemble machine learning, %	72.7	68.5	65.0	75.0	79.6	72.2
Human identification approach^a^, %	64.6	58.0	51.3	36.8	61.9	54.5

^a^Self-reported scales cutoff.

As shown in Table 3, we observed a higher distribution efficiency for ML models than for conventional human identification approaches. More technical details of this simulation are shown in Multimedia Appendix 1.

## Discussion

### Short Summary

**Figure 2:**
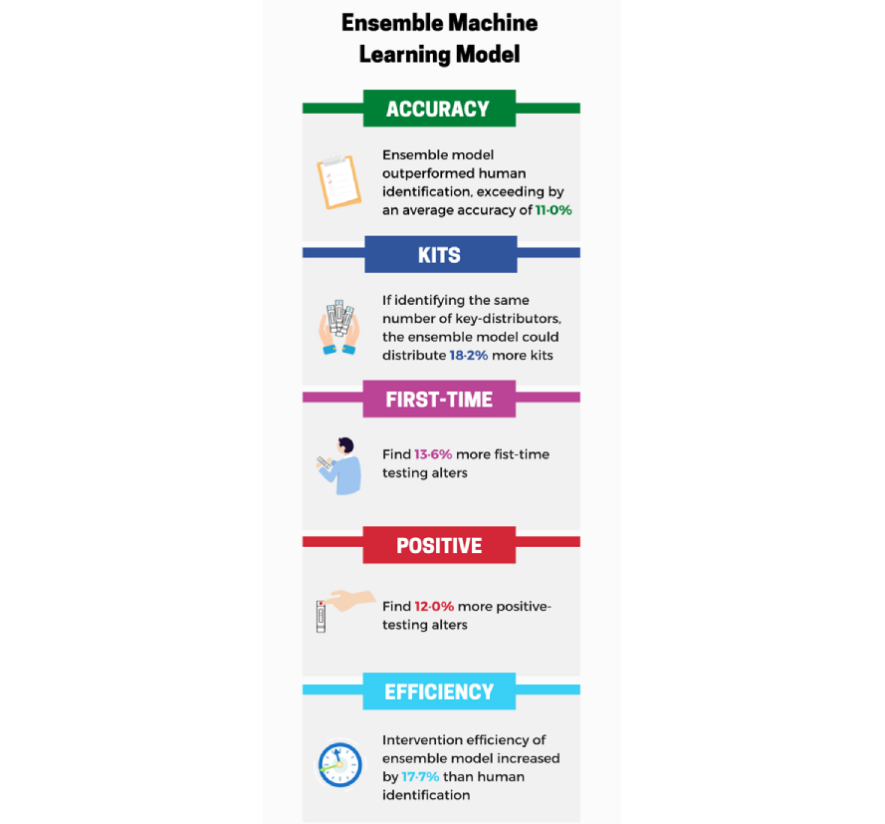
Infographic.

Identifying key influencers for secondary distribution of HIVST kits among Chinese MSM is important. Identification of key influencers who may be more active in the secondary distribution of HIVST kits can potentially expand testing coverage, reach more naïve testers, and help identify undiagnosed people living with HIV. We found that using an ML approach, specifically ensemble learning, was superior to human identification of key influencers (Figure 2).

### Main Findings and Comparison to Prior Work

We found that all 4 ML models outperformed human identification. Our results are consistent with those of other reports that ML models have performed better in other HIV-related identification tasks such as identifying populations at a high risk of HIV [17], identifying HIV-related social media data [30], and identifying people eligible for PrEP [18,31]. This adds to the evidence demonstrating that ML can efficiently identify key influencers within networks compared to other methods.

In addition, we found that key influencers identified by our ensemble learning approach could distribute 18.2% more kits, identify 13.6% more first-time testing alters, and detect 12.0% more undiagnosed people living with HIV than the conventional human identification approach. Regarding why our ensemble learning approach outperformed the human identification approach [14], we believe this may be because the ML algorithms included variables related to 2 key drivers of identification: men's HIV testing and kit application. Self-reported leadership scales are not specifically designed to consider such important predictors. Our data suggest that ML could enhance the accuracy of social evaluation scales for identifying key influencers. Using an ML approach could significantly improve the public health impact of secondary distribution.

Our method offers a potential means to prioritize indexes identified as key influencers in the secondary distribution of HIVST kits using ML. Our novel ensemble ML approach for identifying key influencers in the secondary distribution of HIVST kits can accurately and rapidly classify which indexes are crucial for distributing more kits, promoting testing among novice testers, or detecting more undiagnosed people living with HIV. This is especially significant for low- and middle-income countries where resources for HIV testing services may be limited.

### Limitations

Our study also has several limitations. First, our study had a retrospective modeling design, and we are currently conducting a prospective trial to compare ML and the conventional method [29]. Second, due to the survey content, we only compared our ML approach with 1 type of human identification, namely, self-reported leadership scale cutoffs. Future studies should explore comparisons of the ML approach with other methods of human identification. Third, the sample size (ie, 309 indexes) was relatively small for ML modeling, which could be considered another significant limitation of this study.

### Conclusions and Future Directions

In conclusion, we found that ML using ensemble learning achieved the highest accuracy in identifying key influencers who are more effective at secondary distribution of HIVST kits in China (Figure 2). Therefore, with regard to our future research plans, we are currently implementing this approach in a program involving the distribution of 2000 HIVST kits through a quasi-experimental trial comparing ML identification to scales-based human identification [29]. Our ensemble learning approach can also be generalized to identify key influencers for other HIV prevention and treatment programs.

## Appendix

Multimedia Appendix 1 Technical Details.

Multimedia Appendix 2 Metrics definition, explanation, and formulas in classification performance evaluation.

Multimedia Appendix 3 Predictors selection.

## Abbreviations

AI: artificial intelligence
HIVST: HIV self-testing
ML: machine learning
MSM: men who have sex with men
PrEP: pre-exposure prophylaxis
SD: standard secondary distribution
SD-M: standard secondary distribution with monetary incentives
SD-M-PR: standard secondary distribution with monetary incentives and peer referral

## Data Availability

The training set data for machine learning modeling will be made available to others after obtaining the relevant data sharing agreement. These data can be requested from the corresponding author.
