# Supplementary material for: Identification of Key Influencers for Secondary Distribution of HIV Self-Testing Kits Among Chinese Men Who Have Sex With Men: Development of an Ensemble Machine Learning Approach
Source: J Med Internet Res. 2023 Nov 23;25:e37719. doi: 10.2196/37719 (PMC10704319; doi:10.2196/37719)
Supplement: Multimedia Appendix 2 [file jmir_v25i1e37719_app2.docx]

Multimedia Appendix 2: Table MA2

**Table MA2. Metrics definition, explanation, and formulas in classification performance evaluation**

| Definition | **Meaning and Formula** |
| --- | --- |
| ***TP*** | True Positive |
| ***FP*** | False Positive |
| ***FN*** | False Negative |
| ***TN*** | True Negative |
| ***Accuracy*** | (TP+TN)/(TP+FP+FN+TN) |
| ***Precision*** | TP/(TP+FP) |
| ***Recall (i.e., Sensitivity)*** | TP/(TP+FN) |
| ***F_1_-score*** | 2×Precision×Recall / (Precision + Recall) |

Actually, from mathematical perspective, F1-score is the harmonic mean of Precision and Recall. That is, we can obtain F1-score by 2/F1 = 1/Precision + 1/Recall. Accuracy and F1-score are most widely used in classification tasks as two comprehensive metrics
