# Supplementary material for: Identification of Key Influencers for Secondary Distribution of HIV Self-Testing Kits Among Chinese Men Who Have Sex With Men: Development of an Ensemble Machine Learning Approach
Source: J Med Internet Res. 2023 Nov 23;25:e37719. doi: 10.2196/37719 (PMC10704319; doi:10.2196/37719)
Supplement: Multimedia Appendix 3 [file jmir_v25i1e37719_app3.docx]

Multimedia Appendix 3: Table MA3

**Table MA3. Predictors Selection**

| Selected Predictors | **Variable Dimension** | **Variable Types** |
| --- | --- | --- |
| How many self-test kits do you want to request this time? | HIV testing and kits application | Numeric variable |
| Do you plan to give the kit to others? | HIV testing and kits application | Categorical variable |
| Do you want to get the promotion link of the kit? | HIV testing and kits application | Categorical variable |
| How did you test for HIV the previous time? | HIV testing and kits application | Categorical variable |
| Arm (which groups) | Controlled or intervention group | Categorical variable |
| How frequently do you have sex with her/him? | Social network | Categorical variable |
| What is her/his sexual self-identification (gender) | Social network | Categorical variable |
| In the last 3 months, how many stable male partners did you have? | MSM behavior | Numeric variable |
| In the last 3 months, how many casual male partners did you have? | MSM behavior | Numeric variable |
| In the last 3 months, how frequently did you use the new drugs? | MSM behavior | Categorical variable |
| Overall, how frequently do you discuss HIV/STI related topics with others? | Self-reported leadership scales | Scales variable |
| When you discuss HIV/STI related topics with others in your network, what of the following is the most likely scenario? | Self-reported leadership scales | Scales variable |
